# Effects of Ocean Acidification on the Brown Alga *Padina pavonica*: Decalcification Due to Acute and Chronic Events

**DOI:** 10.1371/journal.pone.0108630

**Published:** 2014-09-30

**Authors:** Teba Gil-Díaz, Ricardo Haroun, Fernando Tuya, Séfora Betancor, María A. Viera-Rodríguez

**Affiliations:** Centro de Biodiversidad y Gestión Ambiental, Universidad de Las Palmas de Gran Canaria, Las Palmas de Gran Canaria, Spain; Griffith University, Australia

## Abstract

Since the industrial revolution, anthropogenic CO_2_ emissions have caused ocean acidification, which particularly affects calcified organisms. Given the fan-like calcified fronds of the brown alga *Padina pavonica*, we evaluated the acute (short-term) effects of a sudden pH drop due to a submarine volcanic eruption (October 2011–early March 2012) affecting offshore waters around El Hierro Island (Canary Islands, Spain). We further studied the chronic (long-term) effects of the continuous decrease in pH in the last decades around the Canarian waters. In both the observational and retrospective studies (using herbarium collections of *P. pavonica* thalli from the overall Canarian Archipelago), the percent of surface calcium carbonate coverage of *P. pavonica* thalli were contrasted with oceanographic data collected either *in situ* (volcanic eruption event) or from the ESTOC marine observatory data series (herbarium study). Results showed that this calcified alga is sensitive to acute and chronic environmental pH changes. In both cases, pH changes predicted surface thallus calcification, including a progressive decalcification over the last three decades. This result concurs with previous studies where calcareous organisms decalcify under more acidic conditions. Hence, *Padina pavonica* can be implemented as a bio-indicator of ocean acidification (at short and long time scales) for monitoring purposes over wide geographic ranges, as this macroalga is affected and thrives (unlike strict calcifiers) under more acidic conditions.

## Introduction

Over the last centuries, atmospheric CO_2_ concentrations have increased due to human activities [1]. As a global net sink, oceans have absorbed almost one third of these anthropogenic CO_2_ emissions [2], causing readjustments in the carbonate chemistry and lowering the pH. This phenomenon was originally known as Ocean Acidification (OA), a term that has been broadened to include other natural events (i.e., increased volcanic activity, methane hydrate releases, long-term changes in net respiration) and anthropogenic causes (i.e., release of nitrogen and sulphur compounds into the atmosphere) [3]. This chemical process has been observed by many oceanic long-term observatories belonging to the OceanSITES programme, such as BATS (Bermuda Atlantic Time-series Study), HOT (Hawaii Ocean Time-series station) and ESTOC (European Station for Time-series in the Ocean at the Canary Islands). Such measurements provide information on the chronic, oceanic pH evolution over time. Calculations have estimated that if CO_2_ emissions continue to rise, global decrease between 0.3 and 0.5 units of surface pH is expected to occur by 2100 based on anthropogenic activities [1], or between 0.06 and 0.32 units according to the latest estimations based on anthropogenic radiative forcing [3]. These values have drawn scientific attention towards their future ecological and physiological impacts, especially on calcified benthic organisms such as corals, echinoderms, gastropods and several calcareous macroalgae [4], [5], [6].

Recently, several studies have been carried out at naturally acidified sites [7], [8], [9], unveiling the effects of vent-induced acidification and the increase in total inorganic C concentrations on the community structure of brown algae, coral-reef associated macroinvertebrates and benthic foraminifera. This field-based research allows the uncovering of long-term effects [4] and combined organisms responses [9], [10], [11]. These studies can be also complemented with mesocosm approaches to target specific cause-effect relationships, despite some limitations (i.e., replication, realism in experiments) [12], [13], [14], [15]. To summarize, this body of research generally points out that OA will cause ecosystem alterations, impacting calcareous but favouring fleshy organisms [6], [16], with the subsequent loss of habitat complexity in the case of calcareous engineering species [9]. The Technical Report of the Secretariat of the Convention on Biological Diversity [17] highlights the main impacts of OA on marine biodiversity using evidences coming from naturally acidified locations, confirming that, although some species may take advantages, biological communities under acidified seawater conditions present less diversity and, in many cases, calcifying organisms are absent. Combined effects of elevated partial pressure of CO_2_ and temperature levels have also been tested, revealing higher decalcification rates in coralline algae [18], loss of coral reef integrity [19] and biomass changes in macroalgal communities [20]. However, little attention has been paid to facultative calcifying organisms and their ecological performances under OA conditions [21], [22], especially to the consequences of long-term (multi-decadal) exposure to rising CO_2_ [9].

High uncertainty still remains regarding the potential impacts of OA on coastal systems; i.e., only few field observations have demonstrated the direct causality of anthropogenic OA on biotic responses [19]. It is suspected that a range of biological processes and functions (other than solely calcification-related issues) are likely to be affected by changes in pH [23]. In addition, OA interacts with other ocean biogeochemical processes (i.e., solubility of trace metals) and environmental changes (i.e., warming and decreasing oxygen levels at a global scale; eutrophication and pollution at local scales) [19], [24]. All of this justifies the need to monitor OA at long-term scales through simultaneous measures of both chemical and biological-effects [23]. However, appropriate bio-indicators that accurately account for the biological effects of OA have not yet been established; several lists of potential organisms are available, related particularly to obligate calcifying organisms [23]. Therefore, there is need for appropriate OA-specific bio-indicators, as well as identification of the biological impacts and future ecological risks due to OA.

A submarine volcanic eruption started in mid October 2011 at *ca.* 1.8 km south offshore El Hierro Island (Canarian Archipelago, eastern Atlantic Ocean). This event caused remarkable changes in the water column chemistry such as in pH_T_ (total scale, at *in situ* conditions), total dissolved inorganic carbon, total alkalinity, pCO_2_, oxygen and nutrient concentrations, as well as in the redox potential by the emission of reduced sulphur and Fe(II) species (specially from November to December 2011) [25], [26]. About 95% of the observed decrease in pH_T_ was related to the emission of CO_2_, contributing to a lesser extent the emission of SO_2_, H_2_S/HS^−^ and the oxidation of dissolved reduced species during the first months [26]. The eruption produced greenish seawater plumes that occasionally extended onshore [25], [27] for several weeks; November 2011 was the most intense period of volcanic activity (lowest pH_T_ level recorded at sea, with a mean decrease of 2.8 units within the first 100 m below the sea level, at *ca*. 2 km away from the volcano) and it officially ended in March 2012 [28]. Changes in the carbonate chemistry of the area immediately affected by the eruption are specified in [26]; i.e., in November 2011, at 5 m above the volcano, values of 7,681.5 µmol kg^−1^ for total dissolved inorganic carbon, total alkalinities of 1,338.0 µmol kg^−1^ and 230,316 µatm of pCO_2_ were registered. This volcanic phenomenon had remarkable effects on the benthic, coastal, communities and was described as an “unprecedented episode of severe acidification and fertilization” [26]; thus, this event may be considered as an example of an acute episode promoting local acidification.

Macroalgae are often considered as indicators of the marine environment health due to their relevant roles in the structuring and functioning of coastal ecosystems [29], [30], [31], [32]. To test the effects of OA we have selected the genus *Padina*, a brown calcified macroalga that facultatively calcifies with extracellular (on the thallus surface) aragonite needles [22], [33] at a rate of 240 gm^−2^ yr^−1^ for the case of subtropical specimens [34]. Diverse members of this genus are distributed widespread in tropical to warm temperate coasts, such as the Macaronesian Islands, Mediterranean Sea, Caribbean Sea, Micronesia and Polynesia [35], [36], [37]. Particularly, *Padina pavonica* (Linnaeus) Thivy plays a significant role as a dominant macrophyte in the Atlantic islands, being a conspicuous member of macroalgal communities in the sub- and intertidal rocky shore systems [38].

Here, we used *P. pavonica* as a biological model to test the effect of natural pH alterations on algal surface thallus calcification (related to OA processes) and its potential sensitivity to acute and chronic OA events. This was accomplished through two complementary approaches: (1) by measuring the effects of the acute OA induced by the volcanic event on the proportion of calcified surface of *P. pavonica*, and (2) by ascertaining the natural calcification trend that this macroalga has followed linked to chronic OA exposure over the last decades using the longest records available for macroalgae: herbarium vouchers.

## Materials and Methods

### Ethics statements

This study was approved by the Canary Islands International Campus of Excellence, which is funded by the Spanish Ministry of Economy and Competitiveness and the coastal land accessed is public land under the Spanish Coast Law. In this study, permits for collections of organisms were not necessary, as seaweeds are unprotected. It was not necessary to have an Animal Care and Use permit according to the national laws.

### Submarine eruption study: acute OA response

**Figure 1:**
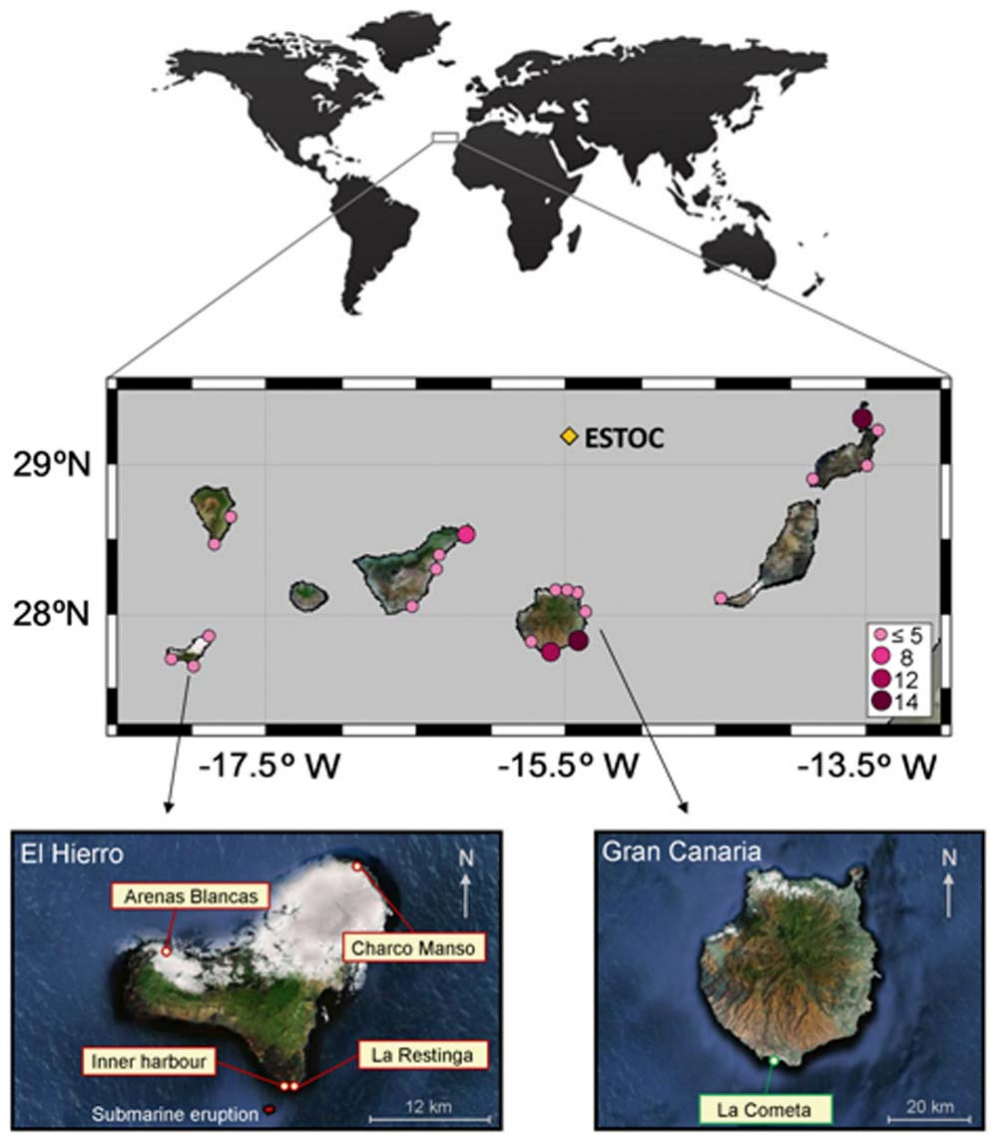
Collection sites of *P. pavonica* in the Canary Islands. Top: herbarium samples denoting the frequency of available sheets (coloured dots). Bottom: sampling sites during the submarine eruption event off El Hierro Island (star) and position of the control site in Gran Canaria Island.

To determine the degree of impact of the submarine eruption south off El Hierro Island on the calcified surface of *Padina pavonica*, n = 17 random samples (individual and unbroken fan-shaped thalli) were collected from the intertidal at five sites (Figure 1; specific coordinates in Table S1). Two sites were adjacent to the eruption (La Restinga and its inner harbour), while the other two were selected further north in the same island (Arenas Blancas and Charco Manso). Additionally, another site (La Cometa) was included - as an external control at more than 200 km from the eruption point - in Gran Canaria Island, as there were doubts as to whether El Hierro Island had any place without the influence of the submarine eruption [21]. Collections of algal material took place at three different periods corresponding to: 24–26^th^ November 2011 (during the greatest eruption activity), 24–26^th^ March 2012 (immediately after the volcanic activity had officially finished) and 2–5^th^ July 2012 (*ca.*>3 months after the official cessation of the eruptive activity). At all occasions, ten to fifteen discrete *in situ* (coastal) measures of seawater temperature (°C) and pH_F_ data (free scale, corrected with temperature) were registered at 0.5–1 m depth by using a calibrated probe coupled to a portable multi parameter HI9829 (Hanna Instruments, USA) during low tide; the probe was dipped into open intertidal pools where the samples were collected at the lower intertidal zone. The calibration of the probe was performed following the manufacturer's instructions with the supplied reagents. Our results present accuracies in temperature and pH_F_ of ±0.15°C and ±0.01, respectively, as well as precisions of ±0.24°C and ±0.02.

Collected samples of *P. pavonica* were frozen at −20°C until laboratory analysis. Once there, samples were thawed, left to dry at room temperature and then digitized using a digital eyepiece camera (MVV3000) coupled to a binocular dissecting microscope. To quantify the percent of decalcified surface of this brown alga, a comparison was made between the total area of the thallus and that belonging to the decalcified zones (Figure S1) by using an image freeware tool (Image J, NIH, USA). This way, only the surface distribution of calcium carbonate, and not its total content on each thallus, was quantified, as this is a non-destructive technique (the original sample is thus conserved). Statistical analyses were carried out with univariate PERMANOVA, testing for significant differences in the mean percentage of decalcified surface (n = 17), pH_F_ and seawater temperature between “Site” (fixed factor) and “Time” (random factor) 2-way ANOVA; *a posteriori* tests resolved pairwise differences between sites for each level of time (months). ANOVAs based on permutations (999 in our case) were used to calculate the significance of P-values. The statistic test (pseudo-F) is a multivariate analogue of the univariate Fisher's F ratio, and in the univariate context the two are identical when using Euclidean distance as the dissimilarity measure [39]. Data satisfied homoscedasticity; thus transformations were not needed [40]. Linear regressions (simple and multiple), performed with Sigma Plot 11.0, tested if pH_F_ and temperature were significant predictor variables of the decalcified percentages.

### Herbarium study: chronic OA response

A retrospective study was done using herbarium sheets from the main official Herbaria in the study area: TFC-Phyc from La Laguna University (http://www.gbif.es/ic_colecciones_in.php?ID_Coleccion=9767) and BCM from the University of Las Palmas de Gran Canaria (http://www.herbariobcm.org/). The surface view of 79 sheets registered as *P. pavonica]* was digitized with an Olympus 700 camera. These sheets belonged mostly to the intertidal zone (86% of the cases) of several sites (Figure 1; specific coordinates and sampled depths in Table S2), from almost a regular record between 1979 and 2012 (21 years of data out of 34). The percentage of calcified surface was quantified by comparing the number of pixels corresponding to calcium carbonate coverage to those shaping the entire thallus (Figure S2), using the Image J software (NIH, USA). We worked with binned data (12 groups of 3 years each) to overcome the uneven distribution of data sets of *P. pavonica*, as some years had many more herbarium sheets than others.

Sea surface temperature (SST) and oceanic pH_T_ values (total scale, at *in situ* conditions) were obtained from ESTOC, a European time series observatory buoy from the EuroSITES and OceanSITES network located at *ca.* 100 km north (Figure 1) from Gran Canaria Island. These data were registered at 1.5 m depth with a Sindemar Mod. SW-03 sensor (pH_T_ values) and a SBE microcat (for SST) (EuroSITES webpage: http://www.eurosites.info/estoc.php). Data were downloaded from the Ocean CO_2_ CDIAC webpage (http://cdiac.ornl.gov/oceans/), providing an available period from 1995 to 2009 (with irregular measurements per year). Statistical analyses were carried out with Sigma Plot 11.0 to test for linear regressions (simple and multiple) between the natural trend of the calcified percentages of the thalli, pH_T_ and SST over time (dependent variables), as well as the relationship between these parameters, taking pH_F_ and temperature as the independent (predictor) variables and the calcified percentages as the dependent variable.

## Results

### Submarine eruption study: acute OA response

**Figure 2:**
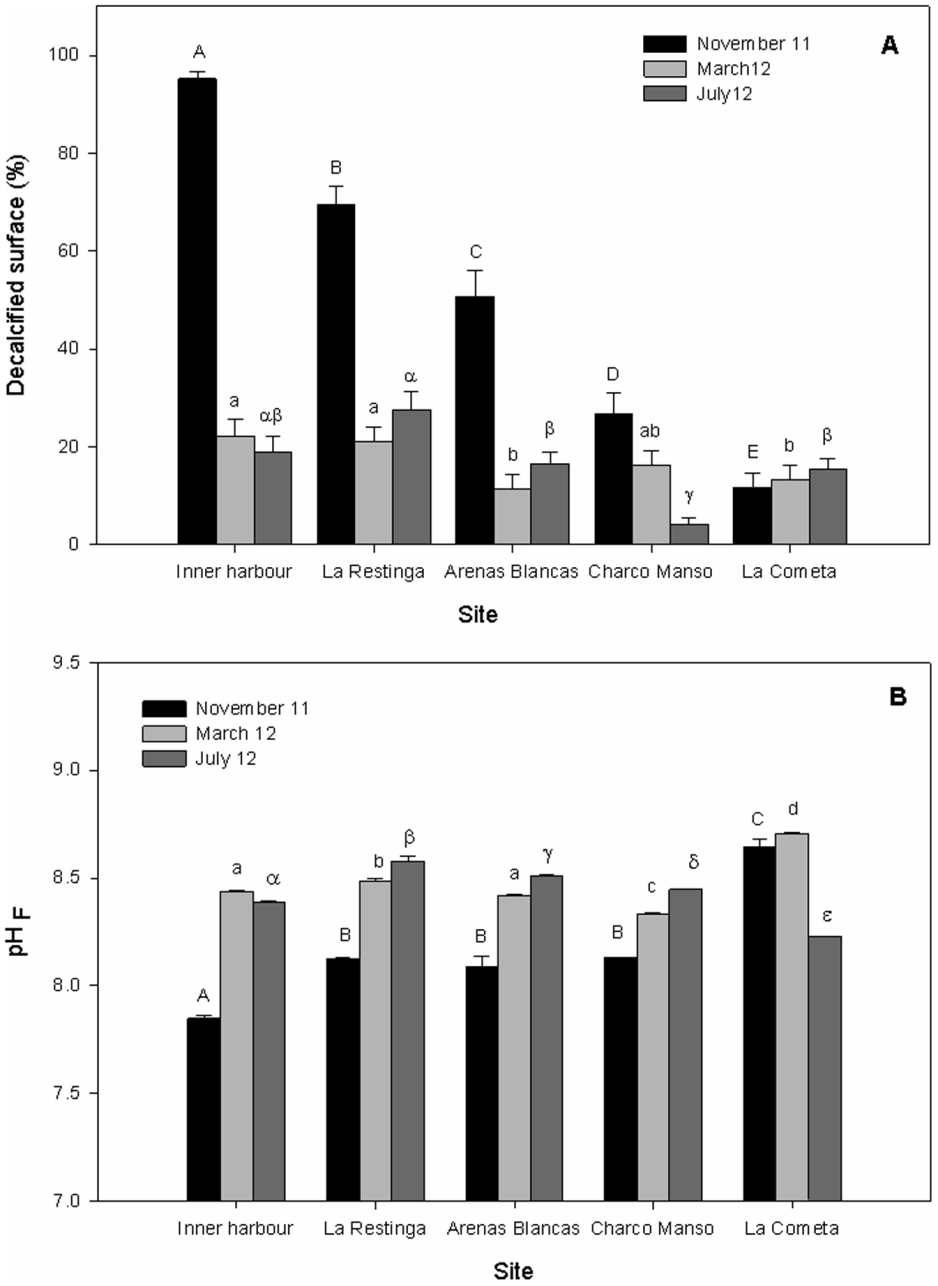
Mean decalcified surface percentages (n = 17) of *P. pavonica* (A) and mean pH_F_ levels (n = 10 to 15) (B) per site and time in an acute OA response. Sites are distributed at increasing distance from the submarine eruption from left to right. The different letters above error bars refer to significant differences (P<0.05) between sites separately for each time (*post hoc* comparisons). Error bars are + SE of means.

The percentage of decalcified surface differed between sites inconsistently through time (2-way ANOVA: Ti×Si, P<0.001, Table 1). In November 2011 (i.e., during the highest eruptive activity), the percentage of decalcified surfaces in *P. pavonica* showed an increasing trend (i.e., more gaps without calcium carbonate) as the distance to the submarine eruption decreased (Figure 2A; Figure S3); the highest decalcification (95.19%±6.32%) was detected in the inner harbour of La Restinga, in contrast to the control (11.76%±11.47%). However, by March 2012 and July 2012, significant differences in decalcified surfaces between sites were attenuated; i.e., similar values in the thalli of *P. pavonica* from El Hierro Island relative to those from the control site (Figure 2A). We have partially published some of these results in a previous study that compares the physiology and photosynthesis of decalcified and calcified thalli of *P. pavonica* [21]. Nevertheless, here we broaden the results to analyze the relationship between thalli decalcification and coastal pH_F_, its variability compared to surface oceanic pH (chronic study) and to test the influence of coastal seawater temperature within this context.

**Table 1:** Two-way ANOVA testing the influence of “Site” (fixed factor) and “Time” (random factor) on the percentage of decalcified surface in *P. pavonica* (n = 17), pH_F_ (n = 10 to 15) and seawater temperature (n = 10 to 15).

	Decalcified surface (%)			Coastal pH_F_			Coastal seawater temp. (°C)		
	MS	F	P	MS	F	P	MS	F	P
**Ti**	32895.48	184.41	0.0002	1.01	17.30	0.0002	20.49	95.51	0.0002
**Si**	10175.62	2.0011	0.1640	0.50	1.35	0.3474	4.51	0.79	0.5446
**Ti × Si**	5084.89	28.51	0.0002	0.37	6.31	0.0002	5.68	26.46	0.0002
**Residual**	178.39			0.06			0.21		

**Figure 3:**
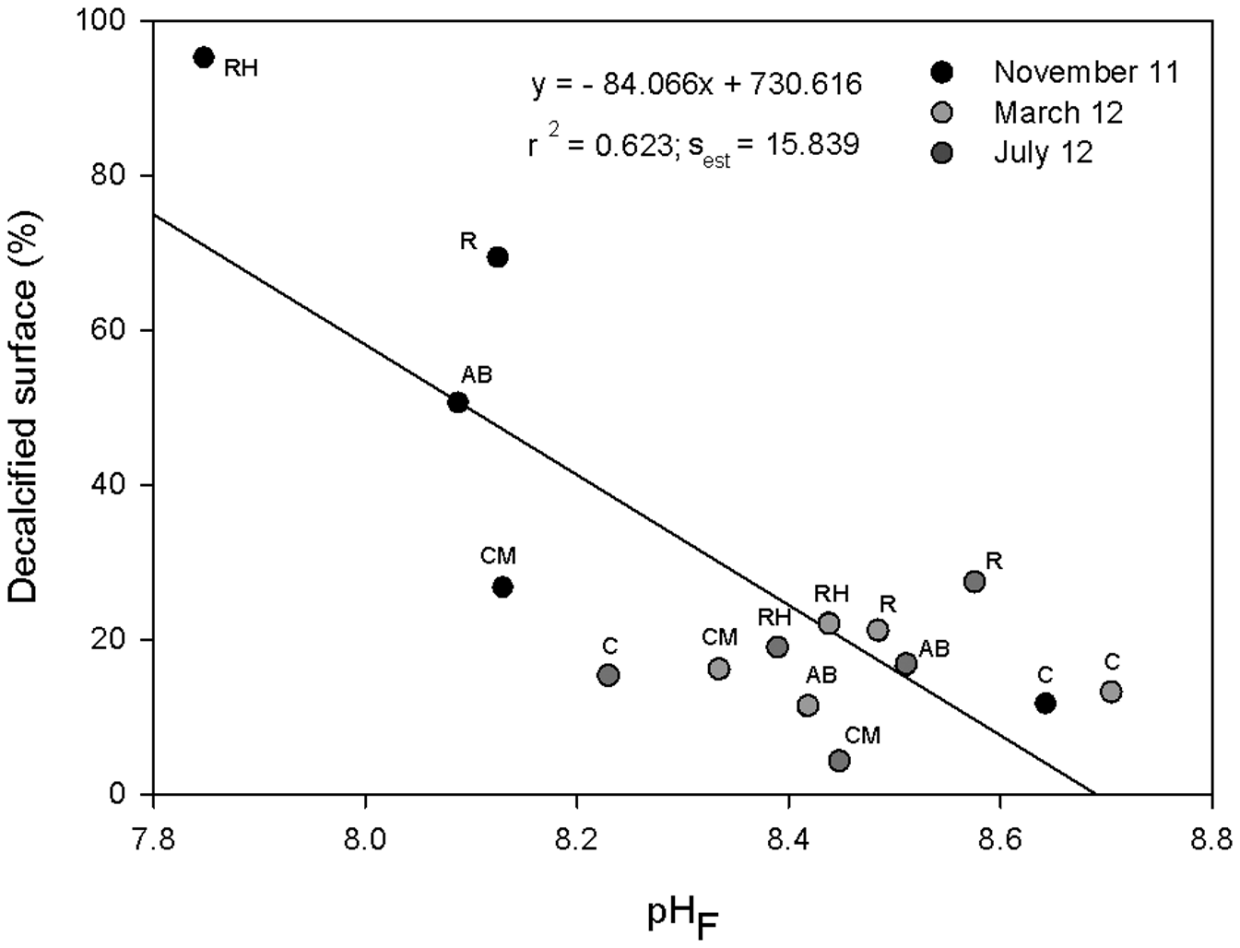
Relationship between decalcification and pH_F_ for an acute OA response. Relationship between mean decalcified surfaces (%) in *P. pavonica* and coastal pH_F_ values for all sites and times (n = 15): (RH) ‘La Restinga harbour’, (R) ‘La Restinga’, (AB) ‘Arenas Blancas’, (CM) ‘Charco Manso’ and (C) ‘La Cometa’ (control). s_est_ stands for standard error of estimate.

Differences in the pH_F_ and coastal seawater temperature between sites also differed inconsistently through time (2-way ANOVA: Ti × Si, P<0.001, Table 1). In November 2011, pH_F_ values significantly decreased at sites located in El Hierro Island relative to the control (Figure 2B), registering 7.38±0.93 units as the lowest mean value (inner harbour of La Restinga). Coastal pH_F_ values at all El Hierro sites in March 2012 and July 2012 rose up to approximately 8.45 units and remained stable; these trends concur with the stabilization of the decalcified values in these months. However, the control site did not show regular pH_F_ values at all times. Changes in pH_F_ were independent relative to the pattern in temperatures at coastal sites, as the latter followed natural trends for the Canarian Archipelago (Figure S4). A multiple linear regression, testing the dependency of mean decalcified percentages to mean pH_F_ and seawater temperature (n = 15), showed that the former was a significant predictor of calcium carbonate coverage on *P. pavonica* thalli (P<0.05, power  = 0.969). Seawater temperature had low multicollinearity with pH_F_ (VIF  = 1.034). These results indicate the independency of seawater temperature relative to pH_F_ and decalcification. Hence, a simple negative linear regression (r^2^ = 0.623; P<0.001, power  = 0.960) was adjusted between mean decalcified percentages and pH_F_ values (Figure 3), showing that a decrease in pH_F_ promoted decalcification in *P. pavonica*. Moreover, it can be observed how the natural pattern present in March 2012 and July 2012 is displaced in November 2011, under the volcanic influence, towards lower pH and higher decalcification percentages.

### Herbarium study: chronic OA response

**Figure 4:**
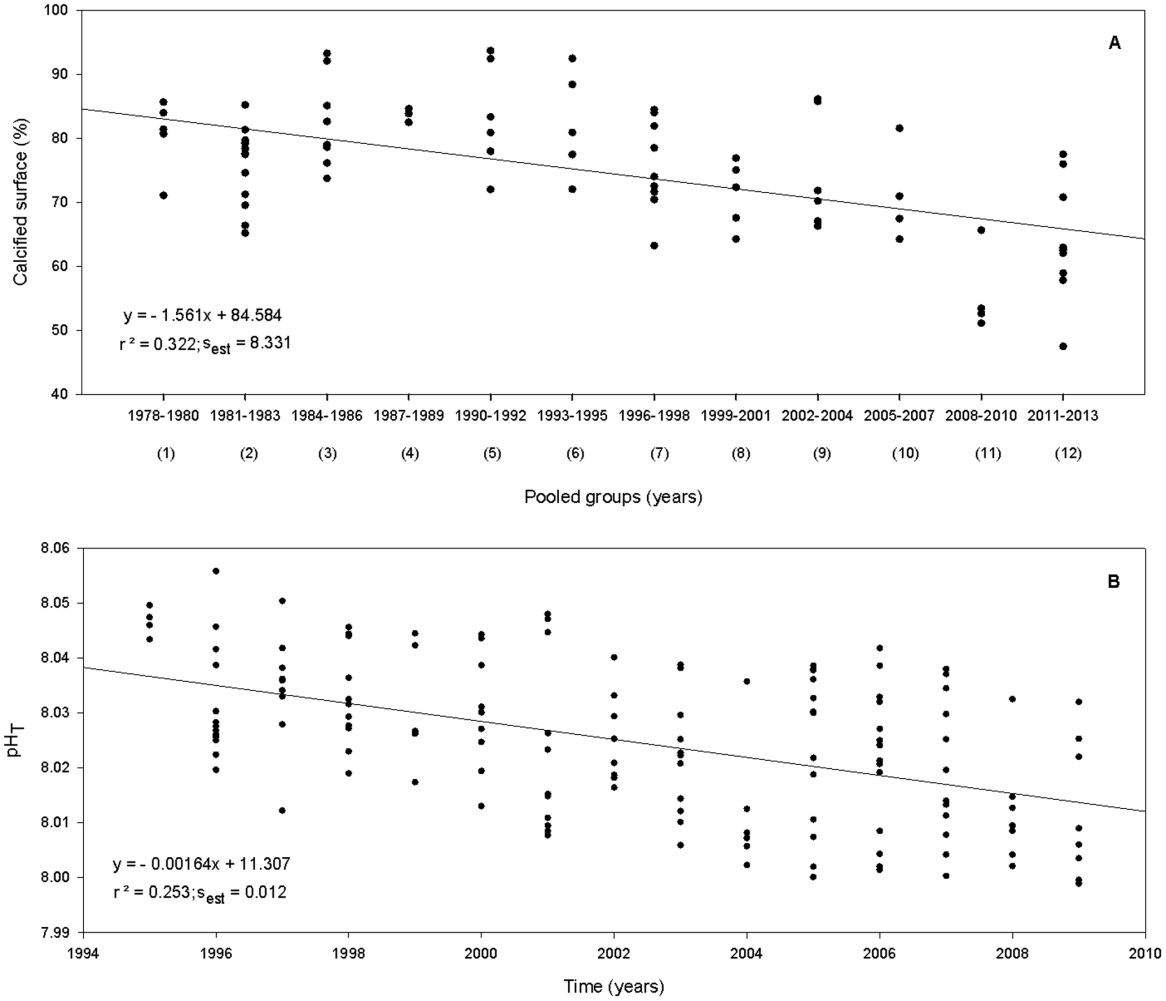
Calcified surface trend of *P. pavonica* thalli from 1978 to 2013 (A) and oceanic pH_T_ levels from ESTOC dataset between 1995 and 2009 (B). The X value in the equation represents the binned group number (1–12) in three year periods. s_est_ stands for standard error of estimate.

Herbarium-derived calcification values (n = 79) showed a decreasing trend in the calcified surface of *P. pavonica* thalli over time (Figure 4A; P<0.001, power  = 1.000). A decrease of 17.17% has been quantified from 1978 to 2013, which yields a mean loss of 0.48% per year.

Oceanic pH_T_ values registered at ESTOC (n = 144) also showed a falling trend over time (Figure 4B; P<0.001, power  = 1.000). The annual variability reflected seasonality (lowest mean pH in winter and highest in summer), a variability that was not noticed in the calcified pattern due to the applied bin. A total decrease of 0.0230 pH units was registered, declining at a mean rate of 0.0015 units per year (1995–2009). Regarding SST measurements through the 1995–2009 period (Figure S5), no specific linear trend was detected (n = 144, P>0.05), although this study had low power analysis (power  = 0.03).

**Figure 5:**
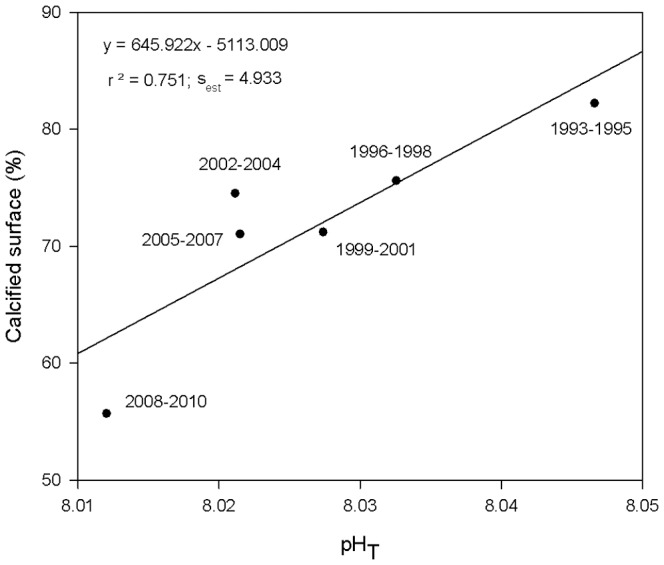
Linear regression between calcified surfaces (%) of *P. pavonica* and oceanic pH_T_ (1993 to 2010). s_est_ stands for standard error of estimate.

When the mean values of calcified percentages, pH_T_ and SST were adjusted for the concurring three year time periods (1993–2010) through a multiple linear regression, no significant pattern was found (n = 6, P>0.05, power  = 0.638). Multicollinearity was low between pH_T_ and SST (VIF  = 3.036). A positive linear regression (r^2^ = 0.75, Figure 5) was then obtained between calcified surface percentages and pH_T_ (P<0.05, power  = 0.628), indicating a positive relationship between the pH and the percent of surface calcification in *P. pavonica* over the last decades.

## Discussion

The submarine volcanic eruption off El Hierro Island, already described at an oceanographic scale [25], [26], affected onshore areas of the entire island in November 2011 (i.e., during the intense eruptive phase), with increasing decalcification on *P. pavonica* thalli related to decreasing pH_F_ values towards the submarine volcano. This result concurs with previous OA models, including field and laboratory studies, where aragonite coral reefs, calcareous algae and epibionts lose CaCO_3_ under more acidic conditions [41], [42], [43]. Furthermore, our results also agree with [22], who observed that CaCO_3_ content in *Padina* spp. is reduced with decreasing pH at volcanic CO_2_ seeps in the Mediterranean Sea and Papua New Guinea.

Importantly, our results showed an apparent sensitivity of *P. pavonica* to acute pH changes as well as resilience, as the alga recovered initial calcification once the intense pH pulses had ceased. This complements the observations provided by [22], where *P. pavonica* thrived (with reduced CaCO_3_) at volcanic CO_2_ seeps. [44] suggest that the effects of OA on calcified species causes increases in dissolution rather than a reduction in the production of calcium carbonate. Moreover, [33] observed a lack of correlation between photosynthesis and carbonate deposition in three species from the genus *Padina* (one of them was *P. pavonica*). Recent studies have already observed that this alga in more acidic environments adapts its physiological performance, behaving as a sun-adapted species instead of a shade-adapted plant as a response to low surface calcium carbonate coverage and thus increased exposure to solar radiation [21]. This could explain its thriving capacity under OA conditions. This is noteworthy, as the herbarium records also showed that *P. pavonica* is sensitive to chronic pH decrease due to OA, denoting its current decalcification trend.

Together with decalcification, *P. pavonica* under low pH conditions, as other macroalgae, decreases its content in phenolic compounds through excretion [21], [45]. Both factors taking place under OA conditions can influence grazing activity, as less phenolic compounds may increase thallus palatability [46] and decalcification can cause a loss in mechanical resistance to herbivory [47]. Thus, the ecological implications of the decalcification of *P. pavonica* may favour increased grazing activity of this macroalga in future scenarios. Nevertheless, according to [48], the ecological performance of *P. pavonica* also depends on its coexistence with other species and how they are affected by OA.

Natural phenomena, such as submarine eruptions or shallow submarine CO_2_ seeps, are considered invaluable environments which provide the closest insight or approach to what might happen in future conditions [16], [49]. In these cases, other parameters (depending on the studied site) such as seawater temperature, usually still show present-day conditions [9]. In this study, the environmental conditions around the volcanic eruption showed pH variations greater than those expected by the IS92a (“business-as-usual”) scenario [1] in the inner harbour of La Restinga (0.57 units of variation) and similar values for the localities of La Restinga and Arenas Blancas (0.41 and 0.38 units of variation, respectively), including no significant variations on the coastal temperature. It is noteworthy that these values also surpass the latest estimations of global ocean surface pH changes (i.e., between 0.30 and 0.32 for the Representative Concentration Pathway 8.5) [3].

In this study, we assumed that pH_T_ trends found in ESTOC observatory act as a baseline of pH values at an insular, onshore, scale. Superimposed on this pH variation, near shore waters may show large short-term pH variability due to events influencing primary production and/or coastal oceanographic anomalies; i.e., upwelling phenomena [19], [50]. This idea cannot be implicitly contrasted here, as no overlapping periods between the open ocean and coastal pH records are available (i.e., no coastal pH data that falls within the ESTOC database period and vice versa for the years 2011–2012). Despite that coastal areas are suspected to display large and uncertain regional and local pH variations [19], our study shows that the sudden decrease in pH in a short period of time induced by the volcanic activity was superimposed on the natural variability of pH. Therefore, it is logical to think that under future pH scenarios of OA (a much slower process), the calcification of *P. pavonica* may follow a trend within a natural range of variability, recovering in favourable conditions and decalcifying under more acidic conditions.

It is also noteworthy that for each study, pH values were measured in different scales, thus their direct comparison is not possible due to their conversion differences, being pH_T_ in general around 0.09 units lower than pH_F_ [51]. However, the chronic pH range seems to be comprised within the observed acute pH range. This observation, together with the positive relation of oceanic pH with calcification percentages, indicates a coincident direction trend with that of the acute values measured during the volcanic event (November 2011). This could support the idea that enriched-CO_2_ volcanic events can act as windows to future pH scenarios.

Despite the evidence of exacerbated effects of combined decreased pH and increased temperatures on calcifiers [52], [53], [54], seawater temperature has not contributed to changes in decalcification patterns. It is possible that, during the volcanic event, the effect of seawater temperature on decalcification of *P. pavonica* was masked by the dramatic pH levels experienced. However, the results from our chronic study suggest that, most likely, there is no direct influence of coastal seawater temperature on calcification. It is possible that the link between *P. pavonica* and the temperature effect is more related to the potential of this alga to spread geographically, as indicated by [55]. For this reason, *P. pavonica* is classified as a climate change affected species for the UK, Wales, Scotland and Ireland [56], [57].

In general, bio-monitors are seen as complementary tools to chemical monitoring programs, as they provide the biological context of the alterations. Monitoring programs and legislation such as the Marine Strategy Framework Directive [58] require bio-indicators to assess the status of the natural environment. In some occasions, they can act as a shortcut to monitoring all the physical-chemical parameters, as long as the potential confounding factors (biological or environmental) are taken into account.

In conclusion, *P. pavonica* is sensitive to acute and chronic environmental pH changes; this suggests that *P. pavonica* could be a suitable bio-indicator of OA on coastal habitats. New research could provide insight on how this calcified macroalga behaves seasonally and under future OA conditions by combining regular *in situ* image (non-destructive) monitoring of calcium carbonate deposition coupled to continuous physical – chemical measurements. This could supply the lack of tools in current monitoring stations that require both chemical parameters and biological effects using a suitable indicator (OA-specific) [19]. Furthermore, given the extensive distribution of this species, this research is applicable to multiple regions, resulting in a wider geographic range monitoring program that could potentially measure and show the direct effects of seawater pH changes in nature. Many questions remain regarding the real biological and biogeochemical consequences of OA for marine biodiversity and ecosystems, as well as the impacts of these changes on oceanic ecosystems and the services they provide [59]. Further direct studies with multidisciplinary approaches will allow more rigorous predictions of OA scenarios and the discovery of its effects on calcifying marine organisms.

## Supporting Information

Figure S1 **Example of the image treatment used to quantify the percentage of decalcified surface on** ***P. pavonica*** **thallus.** (A) Total thallus area. (B) Decalcified areas.
(TIF)

Figure S2 **Example of the pixel quantification method used for the calcified surface of herbarium** ***P. pavonica*** **thallus.**
(TIF)

Figure S3 **Representative images of sampled thalli in El Hierro Island and the control.** (RH) inside ‘La Restinga harbour’, (R) ‘La Restinga’, (AB) ‘Arenas Blancas’, (CM) ‘Charco Manso’ and (C) ‘La Cometa’ (control), both in November 2011 (Nov) and March 2012 (Mar). Note that thalli coming from the inner harbour of La Restinga were almost totally decalcified in November 2011. Graph paper used as scale.
(TIF)

Figure S4 **Mean seawater temperature registered on each site and time for the acute OA response study.** Sites are distributed at increasing distance from the submarine eruption from left to right. The different letters above the error bars refer to significant differences (P<0.05) between sites for each time (*post hoc* comparisons). Error bars are + SE of means.
(TIF)

Figure S5 **SST values registered at ESTOC between 1995 and 2009 (n = 144).** These results show no specific trend over time. s_est_ stands for standard error of estimate.
(TIF)

Table S1 **Geographical position of the sampled sites in El Hierro Island (La Restinga, La Restinga harbour, Arenas Blancas and Charco Manso) and Gran Canaria (La Cometa).**
(DOCX)

Table S2 **Herbarium specifications regarding coordinates (geographical system and UTM) and depth of collection: intertidal (Inter) and subtidal (Sub) areas.**
(DOCX)
