# Supplementary figures and images for: Effects of Ocean Acidification on the Brown Alga Padina pavonica: Decalcification Due to Acute and Chronic Events
Source: PLoS One. 2014 Sep 30;9(9):e108630. doi: 10.1371/journal.pone.0108630 (PMC4182500; doi:10.1371/journal.pone.0108630)

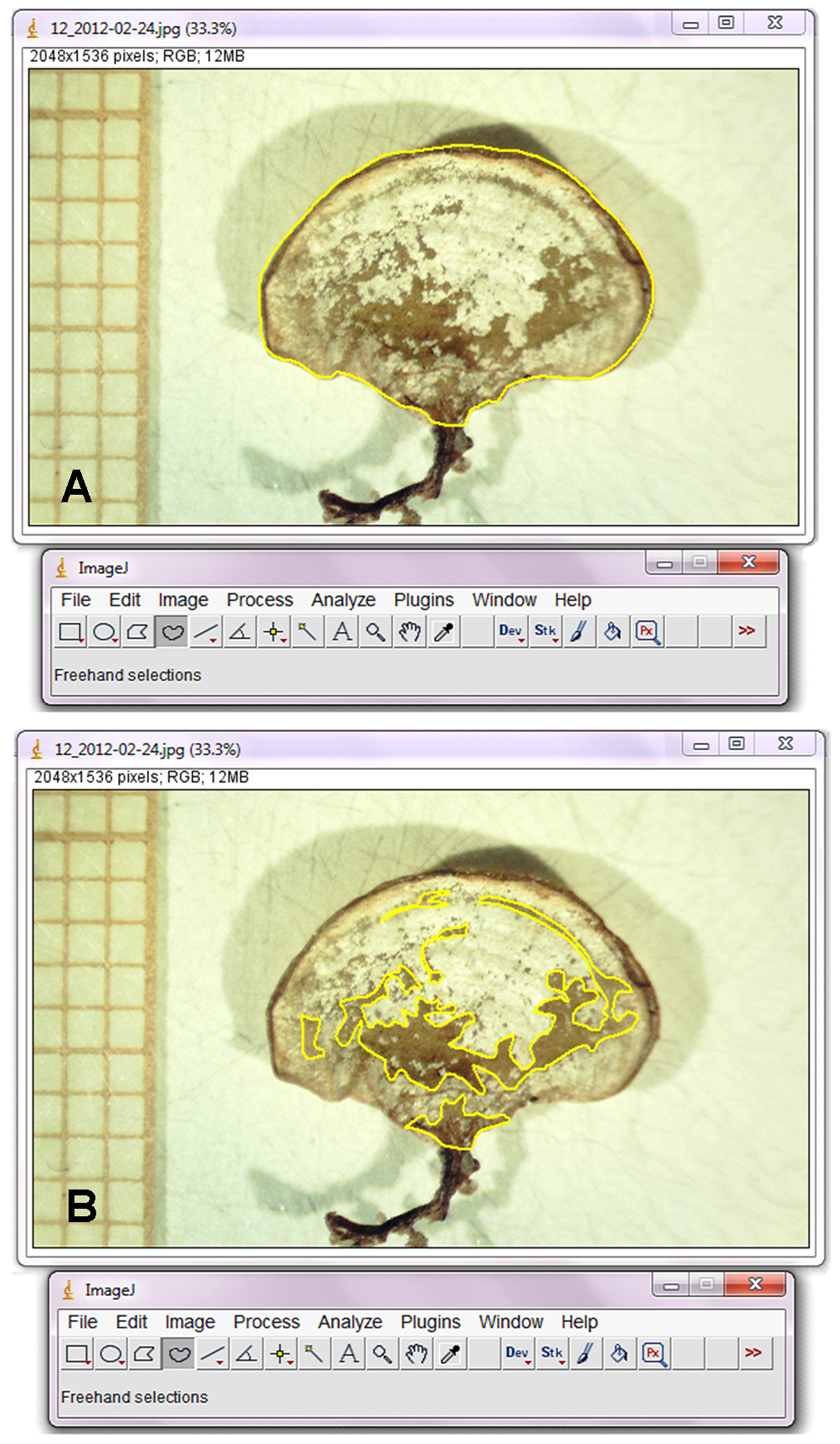

Supplement: Figure S1 — Example of the image treatment used to quantify the percentage of decalcified surface on P. pavonica thallus. (A) Total thallus area. (B) Decalcified areas. (TIF) [file pone.0108630.s001.tif]

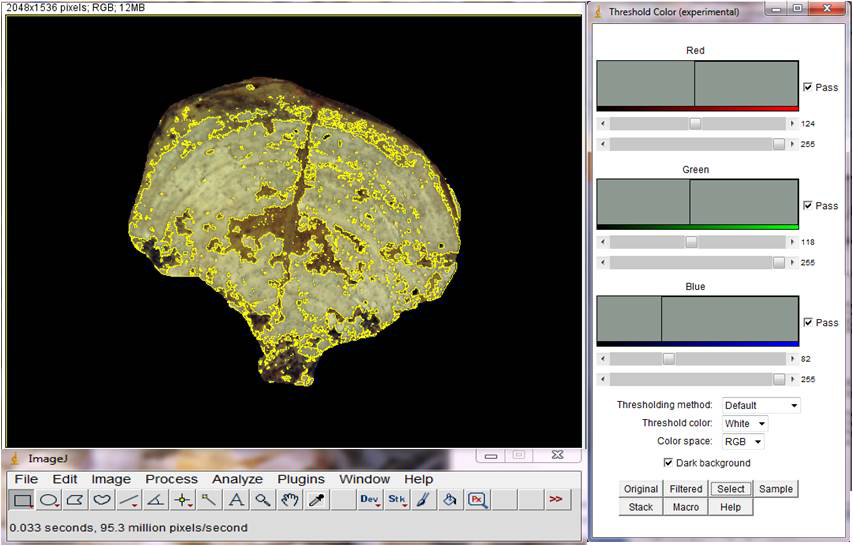

Supplement: Figure S2 — Example of the pixel quantification method used for the calcified surface of herbarium P. pavonica thallus. (TIF) [file pone.0108630.s002.tif]

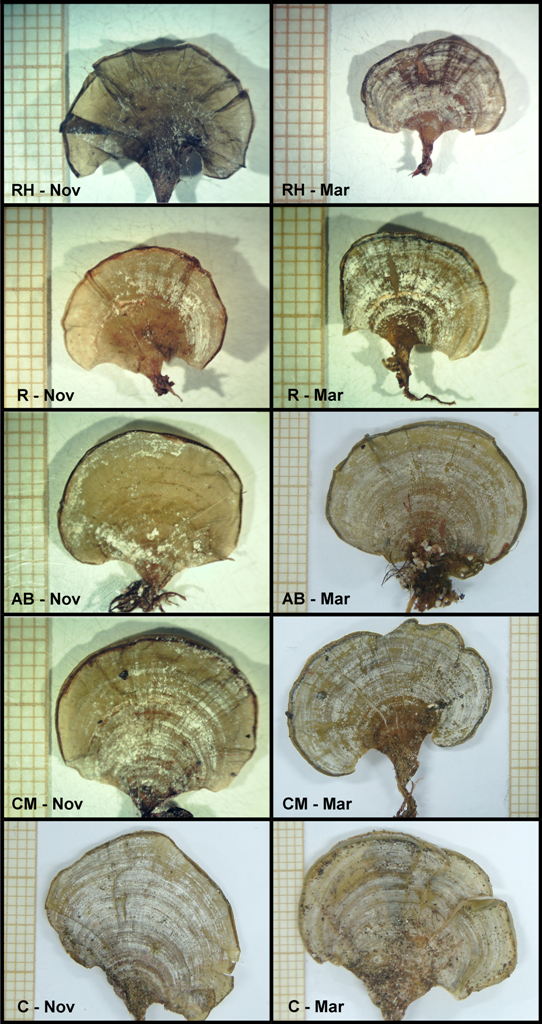

Supplement: Figure S3 — Representative images of sampled thalli in El Hierro Island and the control. (RH) inside ‘La Restinga harbour’, (R) ‘La Restinga’, (AB) ‘Arenas Blancas’, (CM) ‘Charco Manso’ and (C) ‘La Cometa’ (control), both in November 2011 (Nov) and March 2012 (Mar). Note that thalli coming from the inner harbour of La Restinga were almost totally decalcified in November 2011. Graph paper used as scale. (TIF) [file pone.0108630.s003.tif]

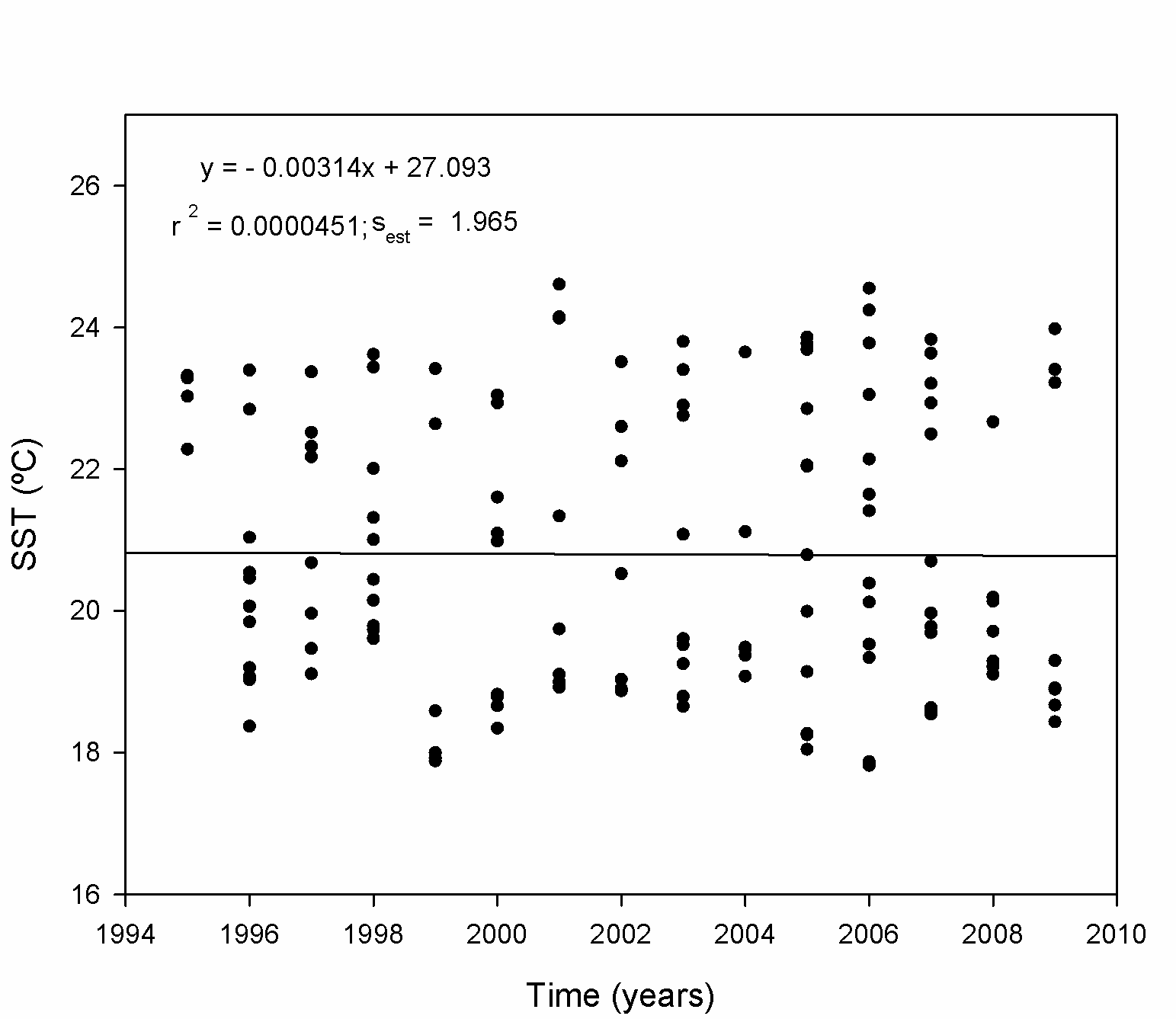

Supplement: Figure S5 — SST values registered at ESTOC between 1995 and 2009 (n = 144). These results show no specific trend over time. sest stands for standard error of estimate. (TIF) [file pone.0108630.s005.tif]
