# Supplementary material for: Effects of Ocean Acidification on the Brown Alga Padina pavonica: Decalcification Due to Acute and Chronic Events
Source: PLoS One. 2014 Sep 30;9(9):e108630. doi: 10.1371/journal.pone.0108630 (PMC4182500; doi:10.1371/journal.pone.0108630)
Supplement: Table S1 — Geographical position of the sampled sites in El Hierro Island (La Restinga, La Restinga harbour, Arenas Blancas and Charco Manso) and Gran Canaria (La Cometa). (DOCX) [file pone.0108630.s006.docx]

**Table S1.**

| **Site** | **Geographical coordinates** | |
| --- | --- | --- |
| La Restinga | 27° 38' 27.37" N | 17° 58' 34.31" W |
| La Restinga harbour | 27° 38' 22.53" N | 17° 58' 59.69" W |
| Arenas Blancas | 27° 46' 04.47" N | 18° 07' 16.06" W |
| Charco Manso | 27° 50' 54.39" N | 17° 55' 24.79" W |
| La Cometa | 27° 44' 47.47" N | 15° 37' 58.54" W |
