# Supplementary material for: Effects of Ocean Acidification on the Brown Alga Padina pavonica: Decalcification Due to Acute and Chronic Events
Source: PLoS One. 2014 Sep 30;9(9):e108630. doi: 10.1371/journal.pone.0108630 (PMC4182500; doi:10.1371/journal.pone.0108630)
Supplement: Table S2 — Herbarium specifications regarding coordinates (geographical system and UTM) and depth of collection: intertidal (Inter) and subtidal (Sub) areas. (DOCX) [file pone.0108630.s007.docx]

**Table S2.**

| **Code** | **Depth** | **Geographical coordinates** | | **UTM** | |
| --- | --- | --- | --- | --- | --- |
| TFC - 8639 | Inter | 28º 57' 35.83" N | 13º 32' 42.33" W | 28R 641803.12 E | 3204373.38 N |
| TFC - 5620 | Inter | 29º 17' 30.37" N | 13º 31'45.29" W | 28R 642611.56 E | 3241191.91 N |
| TFC - 4989 | Inter | 29º 15' 40.77" N | 13º 28' 49.19" W | 28R 647661.83 E | 3237896.42 N |
| TFC - 4710 | Inter | 29º 17' 23.83" N | 13º 30' 27.32" W | 28R 644977.46 E | 3241030.46 N |
| TFC - 4523 | Inter | 29º 15' 47.92" N | 13º 31' 17.12" W | 28R 643634.21 E | 3238097.20 N |
| TFC - 4627 | Inter | 29º 14' 38.08" N | 13º 29' 32.37" W | 28R 646516.40 E | 3235953.30 N |
| TFC - 3488 | Inter | 29º 16' 45.81" N | 13º 29' 43.89" W | 28R 646121.02 E | 3239880.76 N |
| TFC - 4783 | Inter | 29º 15' 05.69" N | 13º 31' 40.95" W | 28R 643023.26 E | 3236756.40 N |
| TFC - 4060 | Inter | 28º 26' 10.46" N | 16º 13' 37.83" W | 28R 379797.64 E | 3151617.94 N |
| TFC - 815 | Sub (12 m) | 28º 17' 52.45" N | 16º 22' 15.14" W | 28R 365578.68 E | 3130966.74 N |
| TFC - 2218 | Inter | 28º 30' 33.47" N | 16º 10' 50.95" W | 28R 384421.84 E | 3154143.40 N |
| TFC - 2263 | Inter | 27º 50' 23.57" N | 17º 56' 50.26" W | 28R 209705.16 E | 3082993.30 N |
| TFC - 2054 | Inter | 28º 19' 41.83" N | 16º 21' 47.63" W | 28R 366358.14 E | 3134304.70 N |
| TFC - 1018 | Inter | 28º 02' 36.80" N | 16º 32' 12.17" W | 28R 348988.90 E | 3102866.41 N |
| TFC - 1013 | Inter | 28º 08' 03.94" N | 15º 26' 39.12" W | 28R 456456.69 E | 3112329.28 N |
| TFC - 1000 | Inter | 28º 00' 20.63" N | 16º 39' 20.91" W | 28R 337191.65 E | 3098942.07 N |
| TFC - 1003 | Inter | 28º 34' 34.63" N | 16º 19' 45.02" W | 28R 370012.27 E | 3161759.77 N |
| TFC - 1002 | Inter | 28º 17' 39.44" N | 16º 22' 30.40" W | 28R 365159.79 E | 3130563.95 N |
| TFC - 1006 | Inter | 28º 01' 53.17" N | 16º 33' 30.18" W | 28R 346807.53 E | 3101661.72 N |
| TFC - 2705 | Inter | 28º 14' 28.31" N | 16º 24' 07.70" W | 28R 362442.79 E | 3124721.80 N |
| TFC - 2709 | Inter | 28º 14' 07.02" N | 16º 24' 23.76" W | 28R 361993.47 E | 3124071.91 N |
| TFC - 426 | Inter | 28º 08' 08.68" N | 15º 26' 27.62" W | 28R 456696.04 E | 3112318.23 N |
| TFC - 634 | Inter | 28º 22' 41.63" N | 16º 21' 38.24" W | 28R 366679.69 E | 3139848.42 N |
| TFC - 708 | Sub (4 m) | 28º 34' 03.38" N | 16º 14' 20.03" W | 28R 378824.91 E | 3160710.58 N |
| TFC - 281 | Inter | 28º 01' 44.21" N | 16º 32' 32.95" W | 28R 348375.75 E | 3101373.88 N |
| TFC - 859 | Inter | 28º 17' 52.40" N | 16º 22' 15.19" W | 28R 365578.54 E | 3130965.62 N |
| TFC - 3128 | Inter | 28º 27' 53.28" N | 17º 51' 34.11" W | 28R 220166.87 E | 3151847.28 N |
| TFC - 110 | Inter | 29º 12' 32.01" N | 13º 25' 42.58" W | 28R 652787.25 E | 3232116.75 N |
| TFC - 5118 | Inter | 28º 59' 47.87" N | 13º 49' 48.86" W | 28R 613943.57 E | 3208172.74 N |
| TFC - 5145 | Inter | 29º 02' 03.60" N | 13º 48' 46.35" W | 28R 615574.96 E | 3212379.82 N |
| BCM - 689 | Inter | 27º 38' 41" N | 17º 59' 47" W | 28R 204328.22 E | 3061463.03 N |
| BCM - 724 | Sub (2-10 m) | 28º 27' 24" N | 17º 50' 50" W | 28R 221174.50 E | 3151121.45 N |
| BCM - 793 | Inter | 27º 42' 20" N | 18º 08' 55" W | 27R 781167.27 E | 3067847.14 N |
| BCM - 1343 | Sub (40 m) | 28º 51' 43" N | 13º 49' 24" W | 28R 614757.75 E | 3193260.68 N |
| BCM - 1458 | Inter | 27º 50' 06" N | 15º 25' 04" W | 28R 458744.48 E | 3078896.32 N |
| BCM - 1716 | Inter | 28º 09' 06" N | 15º 32' 03" W | 28R 447539.84 E | 3114127.24 N |
| BCM - 1945 | Sub (17-25 m) | 29º 23' 00" N | 13º 31' 28" W | 28R 643176.25 E | 3251369.39 N |
| BCM - 2014 | Sub (8 - 12 m) | 29º 23' 08" N | 13º 30' 57" W | 28R 644012.67 E | 3251630.24 N |
| BCM - 2071 | Inter | 29º 24' 49" N | 13º 30' 02" W | 28R 645466.20 E | 3254756.88 N |
| BCM - 3190 | Inter | 27º 49'26" N | 15º 25' 22" W | 28R 458348.52 E | 3077759.48 N |
| BCM - 3312 | Inter | 27º 49' 05" N | 15º 25' 14" W | 28R 458558.99E | 3077139.36N |
| BCM - 3417 | Inter | 27º 50' 06" N | 15º 25' 04" W | 28R 458744.48 E | 3078896.32 N |
| BCM - 3911 | Inter | 27º 49'26" N | 15º 25' 22" W | 28R 458348.52 E | 3077759.48 N |
| BCM - 3913 | Inter | 27º 49'26" N | 15º 25' 22" W | 28R 458348.52 E | 3077759.48 N |
| BCM - 3922 | Inter | 27º 49' 05" N | 15º 25' 14" W | 28R 458558.99E | 3077139.36N |
| BCM - 4218 | Inter | 27º 49'26" N | 15º 25' 22" W | 28R 458348.52 E | 3077759.48 N |
| BCM - 4451 | Inter | 28º 06' 34" N | 14º 29' 26" W | 28R 550038.60 E | 3109404.90 N |
| BCM - 4467 | Inter | 28º 06' 34" N | 14º 29' 26" W | 28R 550038.60 E | 3109404.90 N |
| BCM - 4921 | Sub (9 m) | 27º 45' 24" N | 15º 40' 59" W | 28R 432691.24 E | 3070445.21 N |
| BCM - 6171 | Inter | 27º 57'14" N | 15º 22'38" W | 28R 462576.49E | 3093431.79N |
| BCM - 6572 | Sub (18 m) | 29º 18' 39" N | 13º 31' 38" W | 28R 643024.50 E | 3243337.58 N |
| BCM - 6595 | Inter | 27º 57'14" N | 15º 22'38" W | 28R 462576.49E | 3093431.79N |
| BCM - 6602 | Inter | 28º 39' 08" N | 17º 45' 34" W | 28R 230273.73 E | 3172574.93 N |
| BCM - 6703 | Inter | 27º 49' 05" N | 15º 25' 14" W | 28R 458558.99E | 3077139.36N |
| BCM - 6710 | Inter | 27º 50' 06" N | 15º 25' 04" W | 28R 458744.48 E | 3078896.32 N |
| BCM - 7065 | Inter | 27º 50' 06" N | 15º 25' 04" W | 28R 458744.48 E | 3078896.32 N |
| BCM - 7275 | Inter | 27º 47' 06" N | 15º 30' 07" W | 28R 450549.09 E | 3073506.75 N |
| BCM - 7393 | Inter | 28º 08' 43" N | 15º 35' 43" W | 28R 441531.81 E | 3113450.96 N |
